# Interference and Impact of Dysmenorrhea on the Life of Spanish Nursing Students

**DOI:** 10.3390/ijerph17186473

**Published:** 2020-09-05

**Authors:** Ana Abreu-Sánchez, Javier Ruiz-Castillo, María Dolores Onieva-Zafra, María Laura Parra-Fernández, Elia Fernández-Martínez

**Affiliations:** 1 Department of Nursing, University of Huelva, 21071 Huelva, Spain; abreu@denf.uhu.es (A.A.-S.); javirucastillo@gmail.com (J.R.-C.); 2 Department of Nursing, Physiotherapy and Occupational Therapy, University of Castilla-La Mancha, 13071 Ciudad Real, Spain; mariadolores.onieva@uclm.es (M.D.O.-Z.); marialaura.parra@uclm.es (M.L.P.-F.)

**Keywords:** absenteeism, dysmenorrhea, nursing students, women’s health

## Abstract

Dysmenorrhea is a cause of absenteeism in universities which, in the context of nursing studies, may affect mandatory attendance. Moreover, presenteeism is associated with medication errors, patient falls, and a reduced quality of patient care. This study sought to identify the degree of interference of dysmenorrhea on daily life and its impact on academic performance among Spanish nursing students, and to explore the reasons for presenteeism. A cross-sectional descriptive study was conducted on 261 nursing students. Data were collected using a self-administered questionnaire. The chi square tests, chi-square linear trend, Student’s *t*-test, one-way analysis of variance of polynomial contrasts, and post hoc tests for the bi-variate analysis were used to compare the participants’ responses regarding their type of dysmenorrhea and pain intensity. In addition, a multivariate regression was performed to predict absenteeism. The answers to the open questions were analyzed using thematic content analysis techniques. We observed 62.8% of absenteeism and 92.7% of presenteeism due to dysmenorrhea. Absenteeism was observed to be 3.079 (confidence interval (CI): 95%1.724–5.499; *p* < 0.001) times more likely among women with severe menstrual pain, 2.513 (CI 95%1.314–4.807; *p* = 0.005) times more in those suffering from menstrual nausea and 1.936 (CI 95%1.098–3.411; *p* = 0.022) times more frequent in those suffering from diarrhea. The reasons for presenteeism were grouped into five categories: the pain was bearable, it is not a reason to be absent, others don’t consider it a reason to be absent, responsibility and guilt, and academic consequences. Dysmenorrhea can have a significant impact on academic performance. The concern among students about the academic repercussions and even feelings of guilt and incomprehension from others leads to high rates of presenteeism with potentially negative consequences for patient care.

## 1. Introduction

Dysmenorrhea or menstrual pain is a women’s health issue which affects a large percentage of women of university age [1]. Pain is commonly located in the pelvic region or lower abdomen and is generally chronic and recurrent, coinciding with menstruation, and can often be accompanied by other symptoms, the most frequent being irritability, fatigue, nausea, headaches, and dizziness [2]. There are two types of dysmenorrhea: primary and secondary. Primary dysmenorrhea is associated with menstrual pain for which there is no identified organic cause and which is fundamentally associated with an excess of prostaglandins [3,4,5]. However, secondary dysmenorrhea is associated with an identified organic cause, such as endometriosis [6,7]. Various studies have shown that dysmenorrhea can significantly reduce quality of life and limit daily activities, especially in women with severe pain [8,9]. However, the majority of studies show that women suffering from dysmenorrhea generally do not seek professional medical help and recur to self-medication using non-steroidal anti-inflammatories [10]. This pain relief strategy is often ineffective and may have adverse side effects [11].

During their university education, European nursing students are required to receive both theoretical and practical training, the latter including a minimum of 2300 h of mandatory clinical placements [12]. This implies a meticulous oversight of attendance during internships and therefore universities have implemented rigorous procedures for the management of absences and the ability to make up outstanding hours missed to ensure students meet the minimum hours required. The majority of Spanish nursing students are young women, and research shows that a large percentage of these students suffer from dysmenorrhea [13]. However, there are a limited number of studies which analyze the academic repercussions of dysmenorrhea among nursing students [1]. Only one study of Spanish women has been identified and, although it did address this issue, this was not its primary focus as it merely reported the rates of absenteeism related to this condition [2]. In other countries, a number of studies have analyzed the impact of menstrual pain on absenteeism and/or academic performance, however there is a notable lack of studies on the phenomenon of presenteeism, understood as the practice of attending classes or clinical placements when suffering from severe menstrual pain [14,15], in spite of the fact that evidence shows an increased risk of medication errors, patient falls, and a reduced quality of patient care. Since the majority of nursing students are young women, a group that suffers most from primary dysmenorrhea, they are required to attend clinical placements. However, it has been shown that presenteeism has an impact on the safety of the patients they care for. Therefore, nursing students were considered to be the ideal population to study the interference of dysmenorrhea on their daily lives and academic activities.

The present study aims to identify the degree of interference of dysmenorrhea on daily life and the impact on academic performance among Spanish nursing students (absenteeism, presenteeism, and academic performance), and to identify symptoms that increase the probability of absenteeism as well as to explore the reasons for presenteeism.

## 2. Materials and Methods

### 2.1. Study Design

A total of 261 nursing students suffering from dysmenorrhea participated in a cross-sectional descriptive study.

### 2.2. Settings and Participants

The study sample consisted of university nursing students suffering from menstrual pain. The study included women between the ages of 18 and 35 who reported suffering from menstrual pain (at least three cycles per year and at least one in the last six months [13,16,17]) and enrolled in the Nursing Degree at the University of Huelva during the 2019/2020 academic year. Students who did not meet the inclusion criteria, exchange students, and those who declined to participate were excluded from the study. 

The sample size was not calculated beforehand given that all women meeting the inclusion criteria were invited to participate. The rate of female students who participated was 89.4% (261). Seven students were excluded because they were temporarily on exchange outside the university and five students were excluded as, although they were present at the time of sample collection in the classroom, these were students enrolled at other universities who were temporarily on the faculty. Ultimately, 16 women agreed to participate in the project, however three did not complete it in full. Taking into consideration the absenteeism variable (dichotomous: yes/no) as the main variable concerning the repercussions of dysmenorrhea in the life of university women for this study and the calculation of its proportion based on the previous literature [2], retrospective calculations revealed that the power of the contrast was 0.97.

### 2.3. Data Collection

Data were collected by means of an ad hoc questionnaire with a series of closed-ended questions and an open-ended question designed by the research team, based on previous studies. The closed-ended socio-demographic and anthropometric questions included age, height, weight, living environment. A gynecological question was included to differentiate women with primary and secondary dysmenorrhea. Regarding menstrual pain, dichotomous questions were asked about the presence of a list of circumstances based on the opinion of the research team’s clinicians (full bladder, urination, bowel movements, sexual relations, sitting, walking and weight gain) which may contribute to the appearance or aggravation of their menstrual pain. The impact of dysmenorrhea on daily life was measured on a scale of 0 to 10 (where 0 indicates no impact and 10 absolute limitation) [18] in terms of interference in daily life, attention in class, homework, social activities, sports, work, family relations, sexual relations, and relationships. On a dichotomous level (yes/no), students were asked whether they felt that their concentration in class dropped and whether the pain forced them to have to interrupt their activities and sit down. They also asked about the number of days of presenteeism and absenteeism due to menstrual pain during the last year [2]. In relation to perceived quality of life, the Spanish version of the vertical visual analog scale (VAS) of Euroqol 5D was used [19], ranging from 0 (worst imaginable health status) to 100 (best imaginable health status) to collect information on self-perceived health status, similar to previous studies analyzing this variable among women with dysmenorrhea [8,20,21]. The intensity of menstrual pain was measured using a VAS on a scale of 0 to 10 and interpreted in three categories as in previous studies: mild (1–3), moderate (4–6) and severe (7–10) [13,22,23]. Finally, a question was added regarding the students’ average marks on their four previous exams to evaluate academic performance. The open question was directed only to those women who had attended class and/or internships despite feeling unwell during menstruation; the question was: “Why did you attend classes and/or internships despite feeling unwell/ill with menstrual pain?”.

Students were invited to participate in the study by a teacher in the classrooms of the Faculty of Nursing and a researcher provided information about the study who was present while students filled in the questionnaire in order to clarify any doubts. Given that one of the study variables was absenteeism, the research team handed out the questionnaire on different days and weeks, following the recommendations of Young et al. regarding the ethical issues and methodological challenges of studying absenteeism by means of student classroom surveys [24].

### 2.4. Ethical Considerations

All students voluntarily participated in the study after signing an informed consent form. The project received a favorable report from the Research Ethics Committee of Andalusia (1013-N-19) and was conducted in accordance with applicable data protection regulations and the principles of the Helsinki Declaration. 

### 2.5. Data Analysis

The closed questions of the questionnaire were analyzed quantitatively by means of descriptive analysis. The averages and standard deviation were determined for the quantitative variables and the qualitative variables by frequency and percentages. Chi square tests were used to compare the qualitative variables between the groups according to type of dysmenorrhea and a chi-square linear trend test was used to compare the three groups of participants according to menstrual pain intensity (mild, moderate and severe). To compare the quantitative variables among students with primary and secondary dysmenorrhea a Student’s *t*-test was conducted and to compare the same variables among students according to pain intensity researchers used One-factor analysis of variance (ANOVA) of polynomial contrasts and post hoc tests. A logistic stepwise regression was performed to predict absenteeism from symptoms and menstrual severity. Variables that were significant in previous bivariate analyses were included in the model and adjusted for age, type of dysmenorrhea, and Body Mass Index (BMI). All statistical tests were conducted after verifying the appropriateness of their application. The significance level was <0.05.

The responses to the open question were analyzed by means of a qualitative content analysis [25]. Two researchers read all of the responses to obtain an overview of the results prior to analysis. Two researchers (M.-L.P.-F. and M.D.O.-Z.) independently extracted units of text and assigned labels. They later met and agreed on their findings with a third researcher (A.A.-S.) in the case of any disagreements. The extracted codes were grouped into categories using an analytic framework based on the recommendations of Miles et al. [25]. A fourth member of the research team (E.F.-M.) with extensive training in qualitative analysis reviewed and verified the analysis. The final categories were agreed among the entire research team.

A triangulation method was employed to ensure data reliability with two researchers conducting the initial analysis. The results were also reported to five participants to obtain their overall agreement with the results. 

## 3. Results

### 3.1. Socio-Demographic, Anthropometric and Gynecological Characteristics

Table 1 displays the main socio-demographic, anthropometric, gynecological and quality-of-life perception characteristics self-reported by the participants. The mean age of the participants was 21 ± 2.29 years and the BMI was 22.44 ± 3.36 kg/m^2^. Up to 76.6% lived in an urban environment compared to 23.4% who lived in rural areas. Regarding self-perceived of quality of life, on a scale of 0 to 100 in terms of health status, the mean score was 80.04 ± 11.60. A total of 85.2% participants suffered from primary dysmenorrhea, whereas 14.2% suffered from secondary dysmenorrhea.

### 3.2. Situations That Triggered or Intensified Menstrual Pain

The participants identified situations which triggered or intensified menstrual pain. These were: being seated (80.1%), walking (64.4%), lifting a weight (43.7%), and having a full bladder (43.3%). No differences were detected when comparing students with either type of dysmenorrhea. However, more students with severe pain indicated that their pain appeared or increased in these situations compared with those with moderate or mild pain, as shown in Table 2.

### 3.3. Repercussions of Menstrual Pain on the Daily Life of University Students 

The 261 participants answered questions related to the impact of dysmenorrhea on their daily life. Of these, up to 51.3% reported a lack of concentration in class or work due to this problem. This was the most frequent characteristic among female students with secondary dysmenorrhea (73%) compared to those suffering from primary dysmenorrhea (47.8%) (*p* = 0.004). Up to 68.6% of participants with dysmenorrhea also reported the need to stop and sit at some moment during their period due to the pain; this need was reported by more students with secondary dysmenorrhea (78.4%) than those with primary dysmenorrhea (67%), although the difference was not statistically significant (*p* = 0.166).

Table 3 shows the mean self-reported degree of interference of menstrual pain upon daily life on a scale of 0 to 10 (where 0 indicates the minimum and 10 the maximum). The greatest interference was reported with sexual relations, doing sport and pursuing daily life. Students with secondary dysmenorrhea reported more interference in all the circumstances indicated. 

In relation to the intensity of menstrual pain and its impact on daily life, the lack of concentration in class and clinical placements was reported by more students with severe pain (56.5%), than those with moderate (37.2%) and mild pain (19.2%) (*p* < 0.001). Additionally, more students reported the need to stop and sit down at some moment due to menstrual pain among the group suffering severe pain (70.5%), compared to the groups with moderate (57.4%) and mild pain (34.6%) (*p* = 0.001). As presented in Table 4, students with severe pain reported an average degree of interference in daily life, attention in class, during social activities, while practicing sport, during paid work, in family relations, and their relationship with their partner (*p* < 0.05).

### 3.4. Academic Repercussions of Menstrual Pain

Among all participants, the average mark in the last four exams was 7.61 ± 0.90 out of 10; the averages were similar for students with primary (7.60 ± 0.91) and secondary dysmenorrhea (7.61 ± 0.90) (*p* > 0.05). A comparison of the average marks among the groups of students with different menstrual pain intensity shows an average of 7.66 ± 0.88 for students with mild pain, 7.79 ± 0.91 for those with moderate pain and 7.53 ± 0.89 for those with severe pain (*p* > 0.05).

Regarding presenteeism, 92.7% of participants reported having attended classes or clinical placements with menstrual pain on some occasion during the last year and 62.8% of participants reported having been absent during the same period due to this problem. No significant differences were detected among participants suffering from primary or secondary dysmenorrhea.

The mean number of days absent was 2.61 ± 3.96 in the last year and the mean number of days participants attended while feeling unwell was 17.45 ± 35.11 days during the same period; here again, no significant differences were found among students suffering from primary and secondary dysmenorrhea. However, absences due to menstrual pain were more frequent among participants with severe pain (70%), compared with those with moderate (40.4%) and mild pain (19.2%) (*p* < 0.001). Female students with severe pain were also absent an average of more days (3.46 ± 4.55) than women with moderate (1.56 ± 2.38) and mild pain (0.78 ± 1.87) (*p* < 0.001). Attendance to classes and/or internships when feeling unwell was also more frequent among students suffering from severe pain (95.5%) compared to those with moderate (81.9%) and mild pain (69.2%) (*p* = 0.001).

Among all of those who were absent, less than half consulted a medical professional (44.8%); and of these, some 75.3% continued to be absent. This figure is higher than the 55.2% who did not seek professional help (*p* = 0.001). Of the total number of participants, 44.6% took analgesics for the relief of menstrual pain, of these, 18.3% did not consider these to be effective. Among this group, who did not consider analgesics to be effective, absenteeism was more frequent (85.7%) than among those who did consider them as being effective (67.4%) (*p* < 0.001).

The results presented in Table 5 show that menstrual pain resulting in absenteeism was accompanied by other symptoms such as fatigue, nausea, vomiting, dizziness, headache, diarrhea, and depression (*p* < 0.05).

### 3.5. Multivariate Regression to Predict Absenteeism from Menstrual Symptoms and Intensity of Menstrual Pain

Table 6 shows the results of multivariate stepwise regression in which absenteeism was identified to be 3.079 (confidence interval (CI) 95%1.724–5.499; *p* < 0.001) times more likely in students with severe menstrual pain, 2.513 (CI 95%1.314–4.807; *p* = 0.005) times in those suffering from menstrual nausea and 1.936 (CI 95%1.098–3.411; *p* = 0.022) times more frequent in those suffering from diarrhea.

### 3.6. Reasons for Presenteeism When Suffering Menstrual Pain

The reasons for presenteeism were collected using the open question: “Why did you attend classes and/or internships despite feeling unwell/ill with menstrual pain?”

Responses were grouped into five categories: It is bearable/manageable, it is not a reason to be absent, others do not consider it a reason to be absent, responsibility and guilt for absence and academic consequences.

#### 3.6.1. It Is Bearable/Manageable

Student 3: “Because I feel that I can cope with it.”

Student 37: “I think I can take the pain.”

Student 102: “The pain is not so great that I can’t stand it.”

#### 3.6.2. It Is Not a Reason to Be Absent

Student 9: “I don’t think it’s a good enough reason to miss class.”

Student 17: “Menstruation shouldn’t prevent me from doing my daily activities.”

Student 21: “I can’t miss class because of that.”

Student 48: “Because I don’t think it’s so bad that I must stay home; it’s more important to go to class/practices.”

Student 80: “I can’t be off two days every month because of menstrual pain.”

Student 86: “It’s something I have every month and I can’t miss class; I can’t give up my life because of it.”

Student 99: “… I don’t have the luxury of missing class.”

#### 3.6.3. Other Do Not Consider It a Reason to Be Absent

Student 32: “They don’t think period pain justifies missing class.”

Student 79: “… men don’t think it’s justifiable.”

Student 134: “Because I think they won’t understand if I say I’m missing class because of my period.”

Student 149: “… because they don’t understand that menstrual pain is a reason to miss class.”

Student 201: “Because I can’t miss class for a reason they don’t think is justified.”

Student 232: “… it’s not considered a good enough reason to miss class.”

#### 3.6.4. Responsibility and Guilt for Absence

Student 5: “Because I’m responsible and I don’t like to miss class even if I don’t feel well.”

Student 19: “Because I’m very responsible and work or class is obligatory.”

Student 101: “… I think it’s my responsibility to attend class.”

Student 183: “I don’t feel good about myself if I stay home because of menstruation.”

#### 3.6.5. Academic Consequences

Student 2: “I will fall behind if I don’t attend class.”

Student 13: “Because missing class makes it more difficult to follow and understand the work.”

Student 29: “Because I will be lost in the next class.”

Student 67: “Because if you don’t attend you get an absence and missing class because of menstruation is unjustified and then I lose marks.”

Student 92: “It’s bad for me not to attend.”

Student 153: “So I don’t have to recover lost days.”

Student 174: “… you get lower marks.”

Student 198: “… I decided to go because I’m afraid of the consequences of missing class.”

Student: 213 “A lot of teachers don’t consider menstrual pain a reason to miss class.”

Student 239: “I’m afraid of missing something important in class or have to recover hours of my clinical practicum.”

## 4. Discussion

The present study identified a number of circumstances in daily life which trigger or exacerbate the intensity of menstrual pain, most frequently in female students suffering severe pain. In academic terms, 62.8% reported missing academic work (classes or internships) due to menstrual pain within the last year. Absenteeism was more likely in students with severe menstrual pain, and in those suffering from menstrual nausea and diarrhea. However, no differences were detected between absenteeism and the type of dysmenorrhea. Up to 92.7% of participants reported presenteeism, which was also more frequent among students with severe pain. The reasons for presenteeism were grouped into five categories: the pain was bearable, it is not a reason to be absent, others don’t consider it a reason to be absent, responsibility and guilt, and academic consequences. 

Participants reported that dysmenorrhea interferes in a number of aspects of daily life, especially during sexual relations, when practicing sport and during normal daily activities. This interference in daily life and sport had been reported in previous studies with similar results [1,8,26]. In the case of secondary dysmenorrhea associated with endometriosis, interference with sexual relations due to pain has been studied for decades [27,28,29]. However, our study showed a high degree of interference during sexual relations among students with primary dysmenorrhea. This can have a significant impact on women’s health. However, few studies have been conducted regarding this issue, perhaps due to a social taboo on the subject of sexual relations during menstruation. The present study shows that those with severe pain found this to be more of an interference during the previously activities mentioned than those with mild or moderate pain. Additionally, secondary dysmenorrhea was found to have a greater impact on daily life, the practice of sport, work, and family relations. These results are in line with other studies of women suffering specific pathologies which are manifested with secondary dysmenorrhea, such as endometriosis. These pathologies generally coincide with other symptoms and involve therapeutic treatments which may also have further repercussions on different areas of life, such as repeated surgeries in women with endometriosis [28,30].

Academic performance has been the subject of previous studies, thus, the systematic review and meta-analysis carried out by Armour et al. identified the negative impact of dysmenorrhea on academic performance in both secondary and university education [1]. Our study found slightly lower rates of academic performance among female students suffering severe pain compared to those with mild or moderate pain. However, comparisons of academic performance based on the different types of dysmenorrhea showed no differences, which leads to the conclusion that both types of dysmenorrhea have an equal impact on daily life. Unfortunately, no previous studies on this issue were found to compare our results with. 

Up to 62.8% of participants reported absenteeism due to menstrual pain, similar to the 60.5% reported by nursing students at other Spanish universities [2] but far higher than the 20.1% found in the meta-analysis published in 2019 [1]. This difference is striking and may be due to the fact that the meta-analysis included studies with non-homogeneous sample populations, including secondary school students and university students of various disciplines. Absenteeism due to menstrual pain may be higher in nursing students because clinical placements imply greater physical exertion, more comparable to fulfilling work duties, compared to students attending classes in secondary school or other university disciplines. The high rates of absenteeism point to the need for the academic system to address this issue to avoid hindering the acquisition of skills and competencies by future nursing professionals. 

Among the participants, there were no significant differences in rates of absenteeism, indicating the impact on the daily life of university students with both secondary dysmenorrhea and primary dysmenorrhea, for which there is no identified organic cause. This latter form of dysmenorrhea is most often normalized given there is no objective clinical test for diagnosis and it is often shared by several members of the same family, leading to normalization within the family context [31,32]. These results are a further demonstration of the need for greater community awareness of menstrual health in general and the problem of dysmenorrhea among young women [33].

Seeking professional medical advice for dysmenorrhea, was generally limited to those students who suffered from severe pain and other associated symptoms; these findings are similar to those of previous studies [31,34]. A study conducted at another Spanish university also identified a high level of self-medication without seeking medical advice among women suffering from dysmenorrhea, a common practice predominantly among women with mild or moderate pain [13]. However, it is interesting to note that women who seek professional advice have a higher rate of absenteeism, as do those who find their analgesics to be ineffective and who suffer from other menstrual symptoms. This may be due to the fact that women with more severe pain and additional symptoms tend to be those who most consult medical professionals. However, this also demonstrates that seeking professional help is not enough. In the last decade, several authors have noted the need to further study both the pharmacological and non-pharmacological management of menstrual pain [10,11,35], especially considering that 18% of non-steroidal anti-inflammatory treatments are ineffective for dysmenorrhea, the primary treatment for this condition [36]. There is increasing evidence of the efficacy of non-pharmacological methods for dysmenorrhea, such as aerobic exercise, yoga, taping and transcutaneous electrical nerve stimulation [20,21,37,38,39]. Furthermore, a study conducted in the USA among women suffering from dysmenorrhea identified the reasons why women did not seek medical advice: they believed professionals would be unable to help, they distrusted the available treatments and felt ashamed or fearful of seeking help for this problem [31]. Among the young women participating in this study we found that other menstrual symptoms, such as nausea, vomiting, diarrhea, dizziness, fatigue, headaches, depression, and an inability to concentrate, are more prevalent among women reporting absenteeism. In the participants of this study, absenteeism was more likely among women with nausea and diarrhea. These findings are in line with those of previous studies [2,17], demonstrating the need for professionals to consider not only menstrual pain, but also to adopt a holistic view when evaluating and addressing the problems of each individual women.

Concerning presenteeism, no previous studies were found which analyzed presenteeism in relation to menstrual problems and therefore we were unable to compare our results. However, it should be noted that these future healthcare professionals believed they were acting more responsibly by attending classes and clinical internships when feeling unwell rather than being absent. This contrasts with evidence showing that presenteeism among nurses can pose a greater risk to patient health and safety in terms of, for example, errors in administering medication under these circumstances, reduced quality of care and higher costs [14,15]. Indeed, many students believed that menstrual pain did not justify missing class or clinical training, an attitude that is particularly striking considering these are future healthcare professionals and again highlighting the need for greater awareness and empathy towards the pain of others. Some participants expressed the belief that other people would not consider menstrual pain to be a “good enough” reason to be absent; one replied that men would not understand. These results, drawn from an analysis of the responses to the open question, are in line with the findings of other studies which indicate that menstrual pain is normalized, both socially and within families [31,32]. A number of studies have revealed the educational deficiencies and limitations in addressing sensitive issues, such as the subject of menstruation in our society [1,26,40]. Also of note is the degree of concern students show in relation to the academic consequences of being absent. Despite being a health condition that affects a great number of women, students feel absenteeism due to dysmenorrhea may negatively affect their academic career. These results should be taken into consideration and reveal a real need for education and information within the academic and educational community.

The authors propose that further efforts should be made by policy makers to raise community awareness of the problems of dysmenorrhea, its interference in daily life, and to inform women about the assistance that may be provided by health professionals. At the educational level, individual and institutional responsibility is necessary. In this sense, the mandatory regulations regarding attendance during the clinical training of future health professionals should be revised to comply with European requirements, bearing in mind that absenteeism has a negative impact on the safety of the patient being treated.

### Limitations

When considering the results of the present study, certain aspects must be taken into consideration, such as the cross-sectional nature of this study, the fact that absenteeism and presenteeism were reported retrospectively and that all participants were from the same Faculty of Nursing. However, this is a first approach to the subject and based on these findings it would be interesting to explore this topic in other health science degrees. This research offers insight into current realities and provides a guide for future studies in this area for the development of strategies to minimize the impact of dysmenorrhea.

## 5. Conclusions

Dysmenorrhea, both primary and secondary, interferes with the lives of students who suffer from this condition. Absenteeism associated with dysmenorrhea affects a significant number of nursing students. In many cases, seeking professional medical care or taking analgesic medication is ineffective and therefore an individual approach is required to evaluate different strategies to fully address the problem. In academic terms, it must be noted that students are concerned about the academic repercussions and even experience feelings of guilt and incomprehension from others about this issue. Students believe it is more responsible to attend class and clinical placements even when unwell than to be absent, which carries the risk of a high percentage of presenteeism with possible negative consequences for patient care during clinical practice. These findings emphasize the pressing need for greater awareness and education within the educational community and society in the region of Southern Spain, where this study was conducted.

## Figures and Tables

**Table 1:** Socio-demographic, gynecological and quality of life characteristics.

Characteristics		*n* (%)	M ± SD
Age (years)			21 ± 2.29
BMI (kg/m^2^)			22.44 ± 3.36
Environment	Rural	61 (23.4%)	
	Urban	200 (76.6%)	
Dysmenorrhea	Primary	224 (85.2%)	
	Secondary	37 (14.2%)	
Self-perception of quality of life: health status			80.04 ± 11.60

(M): Mean; (SD): Standard deviation.

**Table 2:** Situations which triggered or intensified menstrual pain in students, categorized by pain intensity (VAS).

Situations which Triggered or Intensified Menstrual Pain		VAS for Pain			Total *n* (%)	*p*-Value ^a^
		Mild *n* (%)	Moderate *n* (%)	Severe *n* (%)		
Pain with a full bladder	No	14 (93.3%)	48 (72.7%)	86 (47.8%)	148 (56.7%)	<0.001 *
	Yes	1 (6.7%)	18 (27.3%)	94 (52.2%)	113 (43.3%)	
Pain when urinating	No	13 (86.7%)	52 (78.8%)	139 (77.2%)	204 (78.2%)	0.452
	Yes	2 (13.3%)	14 (21.2%)	41 (22.8%)	57 (21.8%)	
Pain with bowel movements	No	14 (93.3%)	51 (77.3%)	125 (69.8%)	190 (72.8%)	0.032 *
	Yes	1 (6.7%)	15 (22.7%)	55 (30.6%)	71 (27.2%)	
Pain when having sexual relations	No	13 (86.7%)	54 (81.8%)	134 (74.4%)	201 (77%)	0.131
	Yes	2 (13.3%)	12 (18.2%)	46 (25.6%)	60 (23%)	
Pain when walking	No	8 (53.3%)	30 (45.5%)	55 (30.6%)	93 (35.6%)	0.010 *
	Yes	7 (46.7%)	36 (54.5%)	125 (69.4%)	168 (64.4%)	
Pain when seated	No	7 (46.7%)	12 (18.2%)	31 (17.2%)	50 (19.2%)	0.015 *
	Yes	8 (53.3%)	54 (81.8%)	149 (82.8%)	211 (80.8%)	
Pain when lifting weight	No	13 (86.7%)	39 (59.1%)	94 (52.2%)	146 (55.9%)	
	Yes	2 (13.3%)	27 (40.9%)	85 (47.2%)	114 (43.7%)	

^a^ Chi-square linear trend test; * *p* < 0.05.

**Table 3:** Degree of interference reported by students with primary and secondary dysmenorrhea.

Type of Interference	Type of Dysmenorrhea		Total (M ± SD)	*p*-Value ^a^
	Primary (M ± SD)	Secondary (M ± SD)		
Interferes with daily life	5.0 ± 2.4	5.8 ± 1.9	4.4 ± 2.6	0.038 *
Interferes with attention in class	4.4 ± 2.9	5.4 ± 2.8	3.9 ± 2.9	0.054
Interferes with class work	4.0 ± 3.0	4.9 ± 2.7	3.5 ± 3.0	0.081
Interferes with social activities	4.6 ± 2.9	5.6 ± 2.8	4.1 ± 3.0	0.052
Interferes with sports	5.7 ± 3.2	7.4 ± 2.7	5.4 ± 3.3	0.003 *
Interferes with paid work	2.4 ± 3.1	4.0 ± 3.4	2.4 ± 3.1	0.008 *
Interferes with family relations	2.9 ± 3.1	4.3 ± 3.5	2.8 ± 3.1	0.015 *
Interferes with sexual relations	5.8 ± 3.8	6.8 ± 2.9	5.8 ± 3.6	0.048 *
Interferes with partner relations	3.5 ± 3.3	3.8 ± 3.6	3.2 ± 3.2	0.540

^a^ Student’s *t*-test; * *p* < 0.05.

**Table 4:** Degree of interference self-reported by students with mild, moderate and severe dysmenorrhea.

Type of Interference	VAS for Pain			*p*-Value ^a^
	Mild (M ± SD)	Moderate (M ± SD)	Severe (M ± SD)	
Interferes with daily life	1.69 ± 1.64	3.38 ± 2.01	5.59 ± 2.21	<0.001 *
Interferes with attention in class	1.35 ± 1.70	2.85 ± 2.37	5.04 ± 2.79	<0.001 *
Interferes with class work	1.00 ± 1.60	2.45 ± 2.34	4.69 ± 2.90	<0.001 *
Interferes with social activities	1.58 ± 1.42	2.98 ± 2.51	4.25 ± 2.94	<0.001 *
Interferes with sports	3.08 ± 2.64	4.90 ± 2.96	6.35 ± 3.16	<0.001 *
Interferes with paid work	0.77 ± 1.90	1.54 ± 2.18	3.15 ± 3.35	<0.001 *
Interferes with family relations	1.27 ± 2.36	2.21 ± 2.80	2.91 ± 3.11	<0.001 *
Interferes with sexual relations	4.52 ± 3.23	5.45 ± 3.72	6.23 ± 3.57	0.135
Interferes with partner relations	1.00 ± 1.64	3.05 ± 3.26	3.70 ± 3.29	0.003 *

^a^ One-way analysis of variance (ANOVA) of polynomial contrasts; * *p* < 0.05.

**Table 5:** Menstrual symptoms and absenteeism.

Menstrual symptoms		Absence		Total *n* (%)	*p*-Value ^a^
		No *n* (%)	Yes *n* (%)		
Nausea	No	80 (45.7%)	17 (19.8%)	97 (37.2%)	<0.001 *
	Yes	95 (54.3%)	69 (80.2%)	164 (62.8%)	
Vomiting	No	93 (40.1%)	4 (13.8%)	97 (37.2%)	0.006 *
	Yes	139 (59.9%)	25 (86.2%)	164 (62.8%)	
Fatigue	No	14 (60.9%)	83 (34.9%)	97 (37.2%)	0.014 *
	Yes	9 (5.5%)	155 (65.1%)	164 (62.8%)	
Dizziness	No	77 (44%)	20 (23.3%)	97 (37.2%)	0.001 *
	Yes	98 (56%)	66 (76.7%)	164 (62.8%)	
Headaches	No	44 (44.9%)	53 (32.5%)	97 (37.2%)	0.045 *
	Yes	54 (55.1%)	110 (67.5%)	164 (62.8%)	
Diarrhea	No	46 (47.4%)	51 (52.6%)	97 (37.2%)	0.006 *
	Yes	50 (52.1%)	114 (69.5%)	164 (62.8%)	
Depression	No	38 (47.5%)	59 (32.6%)	97 (37.2%)	0.022 *
	Yes	42 (52.5%)	122 (67.4%)	164 (62.8%)	
Irritability	No	66 (41.3%)	31 (30.7%)	97 (37.2%)	0.086
	Yes	94 (58.8%)	70 (26.8%)	164 (62.8%)	
Insomnia	No	78 (39.4%)	19 (30.2%)	97 (37.2%)	0.186
	Yes	120 (60.6%)	44 (69.8%)	164 (62.8%)	
Inability to concentrate	No	57 (44.6%)	40 (29.9%)	97 (37.2%)	0.012 *
	Yes	70 (55.1%)	94 (70.1%)	164 (62.8%)	

^a^ Chi square tests; * *p* < 0.05.

**Table 6:** Multivariate regression to predict absenteeism from menstrual symptoms and intensity of menstrual pain.

Symptoms and Intesity of Menstrual Pain	OR^a^	CI 95%		*p*-Value
Severe pain	3.079	1.724	5.499	<0.001 *
Nausea	2.513	1.314	4.807	0.005 *
Diarrhea	1.936	1.098	3.411	0.022 *

OR^a^: Odds ratio adjusted for age, type of dysmenorrhea and BMI; CI: Confidence interval; * *p* < 0.05.
